# Supplementary material for: Edible Chitosan Films and Their Nanosized Counterparts Exhibit Antimicrobial Activity and Enhanced Mechanical and Barrier Properties
Source: Molecules. 2018 Dec 31;24(1):127. doi: 10.3390/molecules24010127 (PMC6337635; doi:10.3390/molecules24010127)
Supplement: Supplementary file 1 [file molecules-24-00127-s001.pdf]

Table S1: Tensile strength (TS) properties of chitosan bio-based films prepared from non-sonicated chitosan (NS) and nano-sized chitosan sonicated at increasing times – 5 min($S_{5\ min}$) – and NS/S blends.

c	Mixing ratio	TS / MPa
NS	-	82.2±1.0
NS/$S_{5\ min}$	7:3	61.7±3.7
	1:1	64.2±5.3
	3:7	100.6±7.4
$S_{5\ min}$	-	56.0±6.7

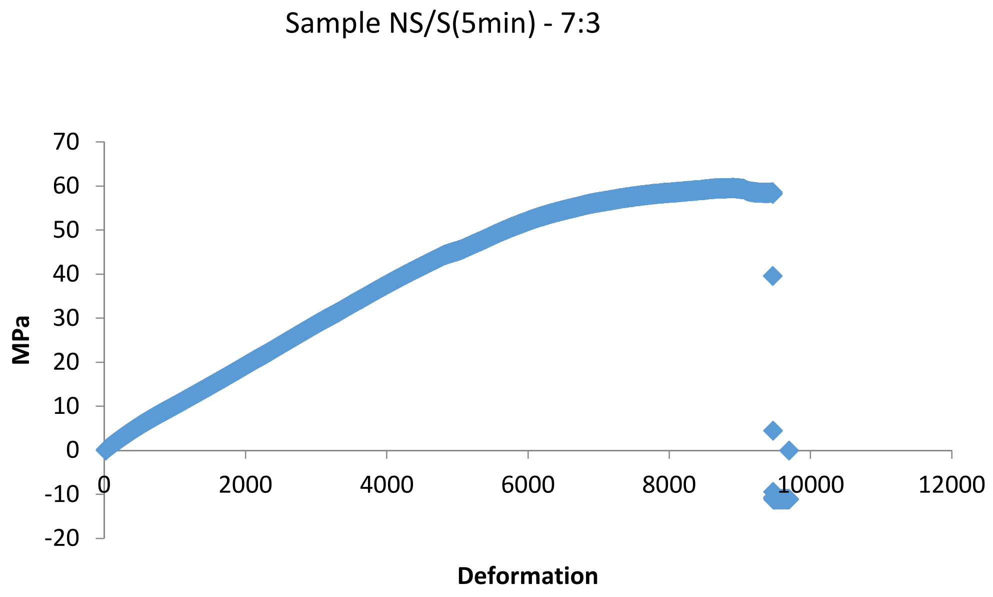

Figure S1: Stress curve for the sample Sample NS/S(5min)—7:3.
